# Female Reproductive Cancers and the Sex Gap in Survival

**DOI:** 10.1001/jamanetworkopen.2026.1256

**Published:** 2026-03-10

**Authors:** Vladimir Canudas-Romo, Wen Su, Emily Banks, Sergey Timonin

**Affiliations:** 1 School of Demography, Research School of Social Sciences, The Australian National University, Canberra, ACT, Australia; 2 Leverhulme Centre for Demographic Science, Nuffield College, University of Oxford, Oxford, The United Kingdom; 3 National Centre for Epidemiology and Population Health, The Australian National University, Canberra, ACT, Australia

## Abstract

**IMPORTANCE:**

On average, females live longer than males. Research on sex differences in longevity has traditionally focused on higher mortality among males; however, the contribution of female reproductive cancers to survival gaps between females and males remains insufficiently quantified.

**OBJECTIVE:**

To assess the female-to-male survival gaps by estimating the contribution of age, birth cohort, and cause of death to sex differences in survival, with a particular emphasis on female reproductive cancers—breast and gynecological cancers—in long-term longevity disparities.

**DESIGN, SETTING, AND PARTICIPANTS:**

This cohort study used population-level mortality data from 20 countries with complete records from 1955 to 2020 from the Human Mortality and World Health Organization Mortality Databases. Data were analyzed from January 2023 to September 2025.

**EXPOSURE:**

Geographic location (Australia, Austria, Belgium, Canada, Denmark, Finland, France, Hungary, Ireland, Italy, Japan, the Netherlands, New Zealand, Norway, Portugal, Spain, Sweden, Switzerland, the UK, and the US).

**MAIN OUTCOME AND MEASURES:**

The primary outcome was the truncated cross-sectional average length of life (TCAL), which incorporates historical mortality information for all birth cohorts alive at a given time. For each country, the sex gap in survival—measured as the difference in TCAL between females and males—was calculated, decomposed, and presented graphically by birth cohort, age, and calendar year.

**RESULTS:**

The analysis encompassed 264.4 million deaths from all causes (119.1 million female [45.1%]; 145.2 million male [54.9%]), including 11.5 million deaths from female reproductive cancers. The sex gap in TCALs ranged from 8.31 (95% CI, 8.28-8.34) years in Hungary to 4.22 (95% CI, 4.20-4.25) years in the Netherlands. Across all countries, females had a survival advantage for major causes of death, except for neoplasms at reproductive ages. In most populations, females aged between 35 and 60 years experienced a consistent cross-cohort excess in cancer mortality compared with males, mainly due to breast cancer and, to a lesser extent, gynecological cancers. Eliminating female reproductive cancers would increase the survival of females and expand the sex gap in TCALs by an estimated mean of 0.77 (95% CI, 0.75-0.78) years, ranging from 0.96 (95% CI, 0.92-1.00) years in Ireland to 0.51 (95% CI, 0.50-0.52) years in Japan.

**CONCLUSIONS AND RELEVANCE:**

In this population-level cohort study of 20 low-mortality countries, females aged 35 to 60 years experienced disadvantage in cancer mortality compared with males—a consistent pattern observed across birth cohorts and over time. These findings underscore the ongoing need for action on the prevention, early detection, and treatment of early-onset female reproductive cancers.

## Key Points

**Question:**

What is the estimated contribution of female reproductive cancers to the differences in survival between females and males?

**Findings:**

In this cohort study of 264.4 million deaths in 20 countries between 1955 to 2020, successive cohorts of females born since the 1930s consistently experienced higher cancer mortality rates than males between ages 35 and 60 years. This female disadvantage was primarily associated with reproductive cancers, particularly breast and gynecological cancers.

**Meaning:**

These findings suggest that female reproductive cancers attenuate total survival among females, including their advantage over males.

## Teaser

This cohort study quantifies differences in survival over time between males and females and examines how female reproductive cancers are associated with these differences.

## Introduction

Females now expect to live longer than males in every country and region of the world.^1^ According to the latest United Nations estimates, the absolute gap in life expectancy (LE) at birth between females and males in 2023 ranged from 0.56 years in Nigeria to 13.30 years in Ukraine, underscoring the complex interplay of biological, social, medical, and contextual factors shaping sex differences in survival.^2^ Empirical evidence from low-mortality countries provides strong support for a persistent survival advantage for females over males, evident as early as the 18th century.^3,4^ Even under extreme external conditions, such as famine or epidemics,^5^ or in more controlled environments with similar lifestyles between the sexes, such as cloistered monks and nuns^6^ and active Mormons,^7^ females have consistently demonstrated higher survival rates than males.

Although the female survival advantage is an almost universal phenomenon, the survival gap varies by age group, calendar year, and populations.^3,8^ The differential contributions of age- and cause-specific mortality to the sex gap in LE offer valuable insights into the nature and possible determinants of this phenomenon. Recent findings suggest that the current male disadvantage in longevity is largely due to higher mortality among males older than 60 years and to specific male subpopulations with a disproportionately higher mortality.^9,10^ In terms of causes of death, cardiovascular diseases, particularly ischemic heart disease and stroke, along with smoking-related cancers and external causes of death, are the main contributors to the sex gap in LE.^11,12,13^ As such, the changing yet persistent excess mortality among males, when compared with their female counterparts within the same country, age range, and birth cohort, can largely be attributed to the differential impact of health and risk-taking behaviors, particularly cigarette smoking, alcohol consumption, and associated health conditions.^14,15,16,17^

Evidence suggests that females generally benefit from an inherent advantage related to biological endowments such as hormonal, autoimmune, and genetic factors.^18^ However, certain hormones, particularly estrogens, are key factors associated with the development and progression of hormone-dependent cancers in females, including breast, endometrial, and certain ovarian cancers.^19,20,21,22^

Most previous research in low-mortality populations has focused on the causes of excess male mortality when seeking to explain the survival gap. In contrast, studies of female disadvantage are rare and typically limited to cross-sectional data at the global level.^23^ In this study, we provide a comprehensive analysis with the goal of estimating the contribution of female reproductive cancers, alongside other major causes of death, to long-term longevity disparities between females and males. To conduct our analysis, we employed the truncated cross-sectional average length of life (TCAL) metric—a relatively recent but increasingly recognized measure in population health research.^24,25,26^

## Methods

This cohort study was exempt from institutional review board review and informed consent was not required because aggregate publicly available deidentified datasets were used in accordance with 45 CFR §46. This study followed the Strengthening the Reporting of Observational Studies in Epidemiology (STROBE) guideline for reporting cohort studies.

### Data Sources

Our analysis was conducted for 20 low-mortality countries with well-established and reliable vital registration systems (eTable 1 in Supplement 1). Age- and sex-specific death rates were obtained from the Human Mortality Database—a widely recognized source of high-quality, harmonized mortality and population data.^27,28^ Age-, sex-, and cause-specific death counts were extracted from the World Health Organization Mortality Database.^29^ We defined female reproductive cancers as breast cancer and gynecological cancers (ie, cervical, ovarian, uterine, vaginal, and vulvar cancers). Several other cause-of-death categories, which account for most of the gap in LE between females and males, were also used for comparison and included neoplasms of the lung, larynx, trachea and bronchus, prostate, and other cancers, along with cardiovascular disease and external causes (accidents, homicides, and suicides). Cardiovascular disease and external causes were selected as illustrative examples of major nonneoplastic mortality groups, with the aim of contextualizing patterns of mortality rather than establishing normative benchmarks for sex equity in care. Details of the mortality data and on the harmonization of the cause-of-death data across *International Classification of Diseases, Seventh Revision (ICD-7)*; *International Classification of Diseases, Eighth Revision (ICD-8)*; *International Classification of Diseases, Ninth Revision (ICD-9)*; and *International Statistical Classification of Diseases and Related Health Problems, Tenth Revision (ICD-10) *are provided in eTable 2 in Supplement 1.

### Statistical Analysis

We employed the TCAL as the primary mortality measure. While conceptually similar to the widely used period LE, TCAL differs in that it incorporates historical mortality information for all birth cohorts alive at a given time, rather than being based solely on current-period mortality rates. The TCAL metric was introduced and described in detail elsewhere.^24,30^ A brief overview of the metric is also provided in the eAppendix in Supplement 1 and an illustration of the data it incorporates is in eFigure 1 in Supplement 1 as a Lexis diagram (a visual representation with ages in the vertical axis, birth cohorts in diagonal lines, and calendar years in horizontal axis). From a public health perspective, this approach enables the identification of birth cohorts and age groups that experienced periods of higher or lower historical mortality, including from specific causes of death.

In this study, we estimated TCAL for each country and sex using mortality data series for a 65-year period, from 1955 to 2020 (country-specific variations in data availability are presented in eTable 1 in Supplement 1). Sex differences in TCAL were subsequently decomposed and visualized using Lexis surfaces.^24^ We report 95% CIs of TCALs to convey sampling variability around point estimates. Confidence intervals were calculated for all measures through a stratified bootstrap procedure (1000 iterations). In each iteration, conditional on age and calendar year, cause-specific death counts were resampled using a multinomial distribution, and the TCAL decomposition was computed for all causes of death (for more details see the eAppendix in Supplement 1). All analyses were performed from January 2023 to September 2025 in R version 4.4.2 (R Project for Statistical Computing) and R Studio version 2025.05 (Posit PBC).^31^

## Results

This cohort study included 20 low-mortality countries (Australia, Austria, Belgium, Canada, Denmark, Finland, France, Hungary, Ireland, Italy, Japan, the Netherlands, New Zealand, Norway, Portugal, Spain, Sweden, Switzerland, the UK, and the US) representing some of the longest-living populations worldwide. The analysis used national mortality data and encompassed 264.4 million deaths from all causes, which were registered in these study countries between 1955 and 2020, including 119.1 million female deaths (45.1%) and 145.2 million male deaths (54.9%). The number of deaths from female reproductive cancers accounted for 11.5 million deaths. In total, 42.9 billion person-years of follow-up were accrued (see eTable 3 in Supplement 1 for detailed comparisons).

### Cross-National Variation in All-Cause TCAL and the Corresponding Sex Gaps

**Figure 1.**
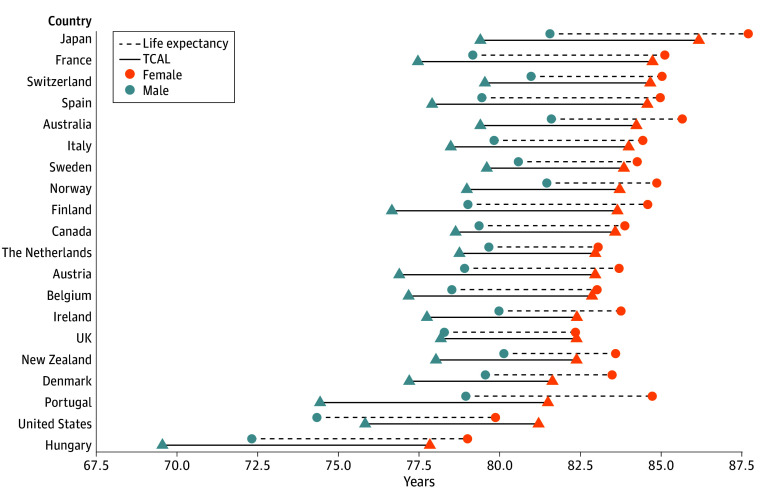
Line Graph of Female and Male Truncated Cross-Sectional Average Length of Life (TCAL) and Life Expectancy at Birth in Selected Low-Mortality Countries TCAL was calculated using mortality data for 1955 to 2020; TCALs for New Zealand and Portugal correspond to the periods 1955 to 2018 and 1955 to 2019, respectively, due to data availability. Life expectancy was estimated for 2020. Countries are ordered by descending female TCAL values, from the highest (Japan) to the lowest (Hungary). Data used are described in eTable 1 in Supplement 1. The exact values of TCAL and life expectancy are presented in eTable 3 in Supplement 1.

Figure 1 shows the all-cause TCAL and LE at birth for females and males, ordered from highest to lowest female TCAL. As expected, sex-specific TCAL values for 1955 to 2020 differed from LE in 2020 because the latter did not capture the historical survival experiences of the birth cohorts alive at that time. In all the countries, except the US (both sexes) and the UK (females), LE was higher than TCAL reflecting sustained improvements in mortality over recent decades. From a cohort mortality perspective, US and UK females exhibited higher longevity as measured by TCAL than by period LE observed during the first year of the COVID-19 pandemic (see eTable 4 in Supplement 1 for detailed comparisons).

In all countries, females had a clear survival advantage over males. The highest female TCAL was in Japan (TCAL, 86.21 years; 95% CI, 86.16-86.26 years). For males, the highest and comparable TCAL values were observed in Sweden (TCAL, 79.62 years; 95% CI, 79.43-79.81 years), Switzerland (TCAL, 79.56 years; 95% CI, 79.34-79.78 years), Australia (TCAL, 79.42 years; 95% CI, 79.28-79.57 years), and Japan (TCAL, 79.41 years; 95% CI, 79.36-79.47 years). Hungary had the lowest TCAL for both females (TCAL, 77.85 years; 95% CI, 77.66-78.02 years) and males (TCAL, 69.54 years; 95% CI, 69.35-69.73 years) and the largest observed survival gap.

The sex gap in all-cause TCAL varied substantially across countries, ranging from a maximum of 8.31 (95% CI, 8.28-8.34) years in Hungary to a minimum of 4.22 (95% CI, 4.20-4.25) years in the Netherlands. When assessed using LE in 2020, the magnitude of the sex gap was smaller in all countries, reflecting historically larger sex differences in mortality—characterized by higher levels and slower declines in male mortality compared with female mortality—over the period captured by TCAL (see eTable 4 in Supplement 1 for exact values).

### Cause-Specific Estimated Contributions to the Survival Gap in TCAL for the US and Japan

**Figure 2.**
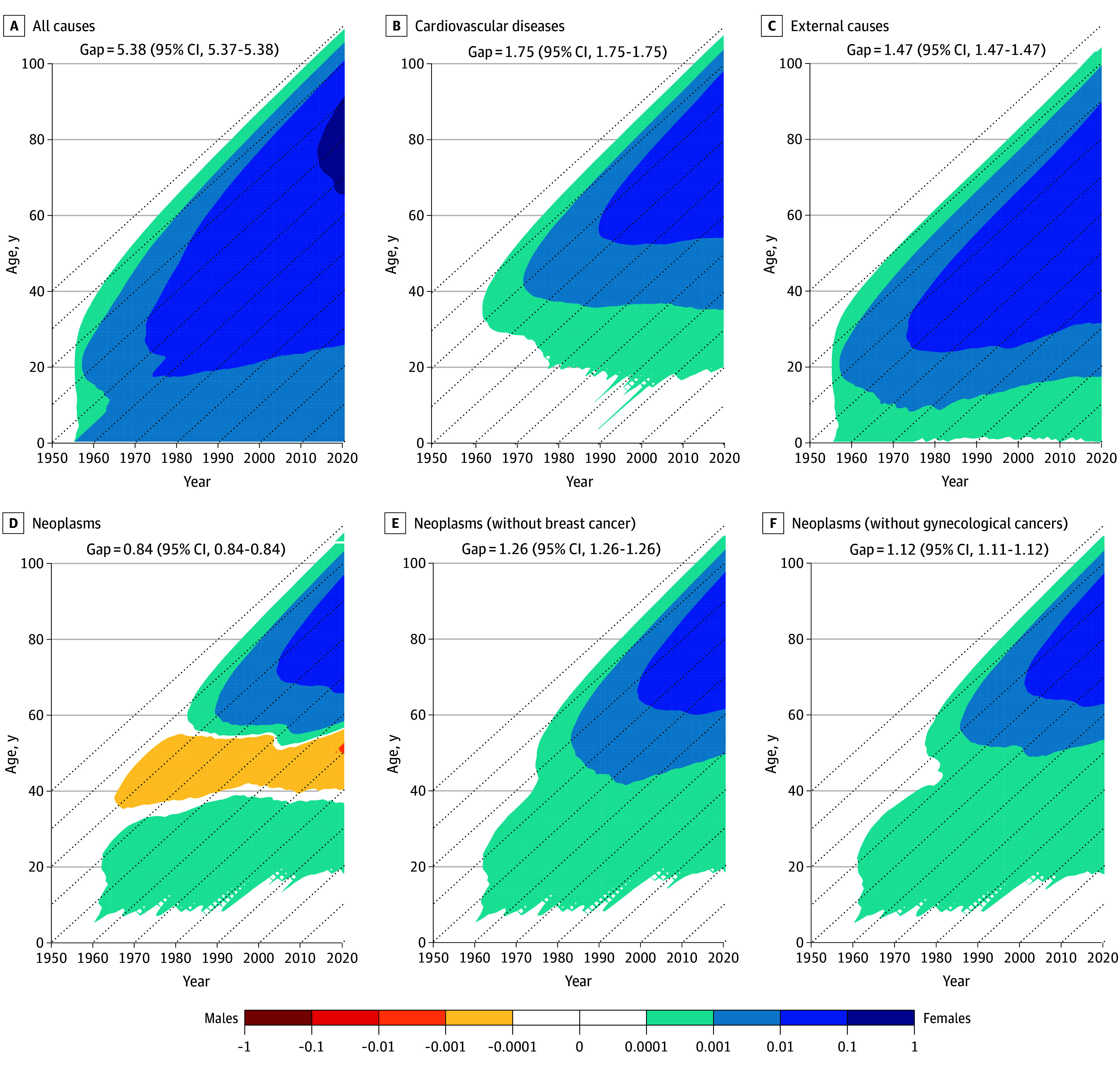
Lexis Surfaces of Sex Differences in Survival and the Contributions of Causes of Death, US, 1955-2020 Sex differences were calculated by subtracting truncated cross-sectional average length of life for males from that of females. Diagonal dashed lines represent birth cohorts. Blue colors indicate female survival advantage and yellow and red colors indicate male advantage. Data used are described in eTable 1 in Supplement 1.

Figure 2 shows the findings regarding the US survival gap for all causes of death and separately for major causes, presented across different Lexis surfaces (a visual representation that enables the examination of the sex-specific survival disparities across ages, birth cohorts, and calendar years). The Lexis surface in Figure 2A shows the total (all-cause) sex difference in TCAL of 5.38 (95% CI, 5.37-5.38) years. The other Lexis surfaces (Figure 2B-D) show the contributions of the 3 major causes of deaths to this gap: cardiovascular diseases (TCAL, 1.75 years; 95% CI, 1.75-1.75 years), external causes of death (TCAL, 1.47 years; 95% CI, 1.47-1.47 years), and neoplasms (TCAL, 0.84 years; 95% CI, 0.84-0.84 years).

Females in the US across all birth cohorts exhibited a survival advantage over males, especially those born in the 1940s, as indicated in Figure 2A. This advantage was consistently observed for cardiovascular diseases (Figure 2B) and external causes of death (Figures 2C), with the notable exception of neoplasms (Figure 2D). A distinct and stable age pattern was evident in this Lexis surface, as shown by a horizontal yellow band. In other words, each successive cohort of females, born since at least 1930, experienced an excess in cancer mortality compared with males between the ages of 35 and 60 years.

To further explore this phenomenon, we estimated the contribution of female reproductive cancers to the magnitude of the survival gap in TCAL (Figure 2E-F). In the case of the US, the elimination of breast cancer would increase the survival gap for neoplasms from 0.84 (95% CI, 0.84-0.84) years to 1.26 (95% CI, 1.26-1.26) years, while eliminating gynecological cancers would widen the gap to 1.12 (95% CI, 1.11-1.12) years. Eliminating both groups of female reproductive cancers would have added 0.70 (95% CI, 0.69-0.71) years to the overall sex gap in TCAL, thereby strengthening the female survival advantage. These Lexis surfaces show that female reproductive cancers contributed most significantly to the female disadvantage in overall cancer-related survival relative to males.

**Figure 3.**
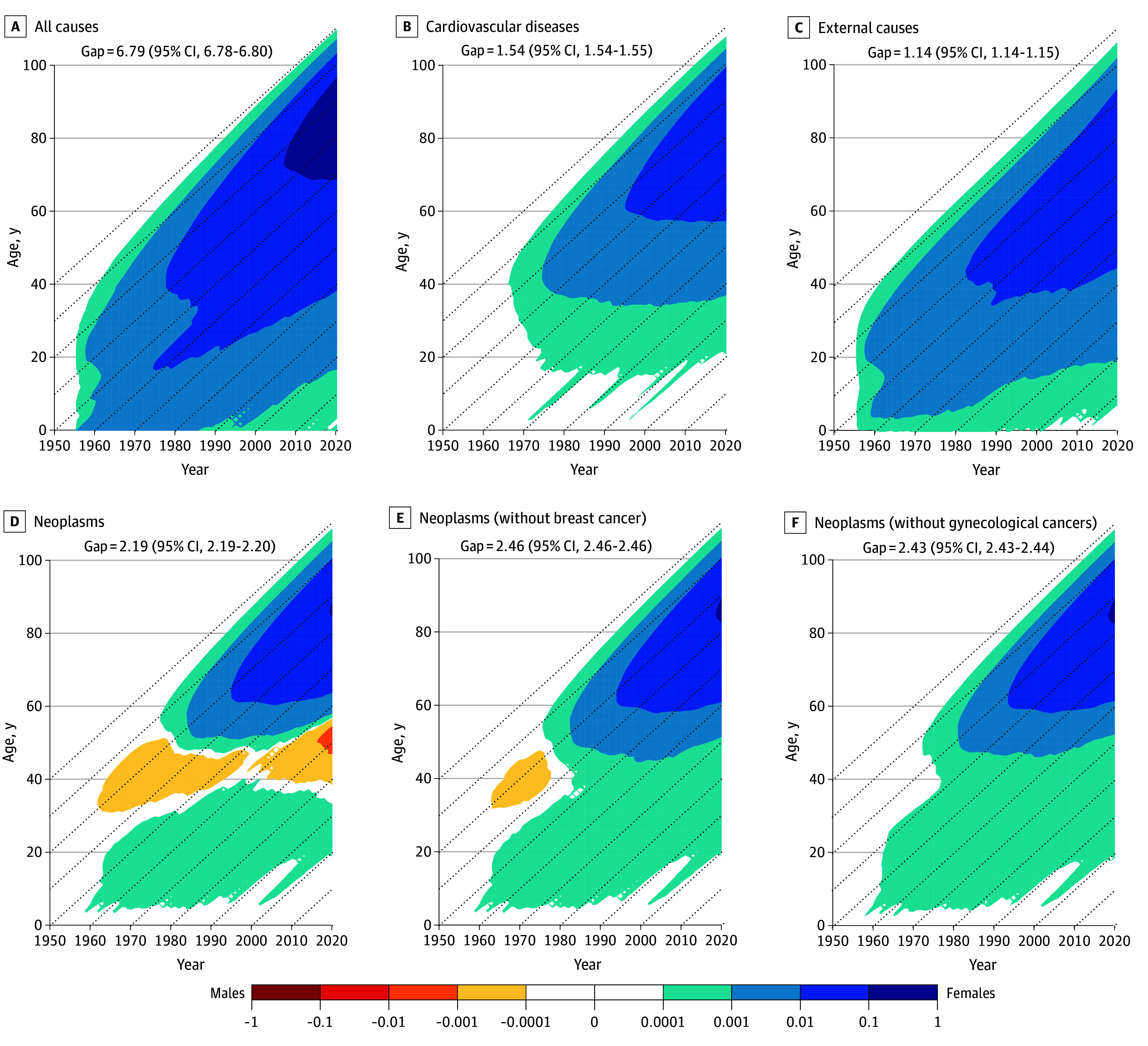
Lexis Surfaces of Sex Differences in Survival and the Contributions of Causes of Death, Japan, 1955-2020 Sex differences were calculated by subtracting truncated cross-sectional average length of life for males from that of females. Diagonal dashed lines represent birth cohorts. Blue colors indicate female survival advantage and yellow and red colors indicate male advantage. Data used are described in eTable 1 in Supplement 1.

Figure 3 presents the results for Japan, the country with the highest female TCAL. Despite their longevity advantage, Japanese females experienced a disadvantage in terms of neoplasm survival at reproductive ages compared with their male counterparts. Breast and gynecological cancers accounted for one-quarter of a year each, with their removal increasing the sex gap in TCAL by one-half of a year from 2.19 (95% CI, 2.19-2.20) years to 2.70 (95% CI, 2.69-2.72) years.

The eResults in Supplement 1 show the Lexis surfaces for the other countries included in the analysis. The female survival disadvantage from neoplasms at young-middle ages was observed in each of the examined countries, with less extent in France, Hungary, Italy, Portugal, and Spain. Females in the Scandinavian countries (Denmark, Norway, and Sweden) and English-speaking countries (Australia, Canada, Ireland, New Zealand, and the UK) were particularly affected by this, with a lesser impact in the US.

### Female Reproductive Cancers and the Survival Sex Gap Across Countries

**Figure 4.**
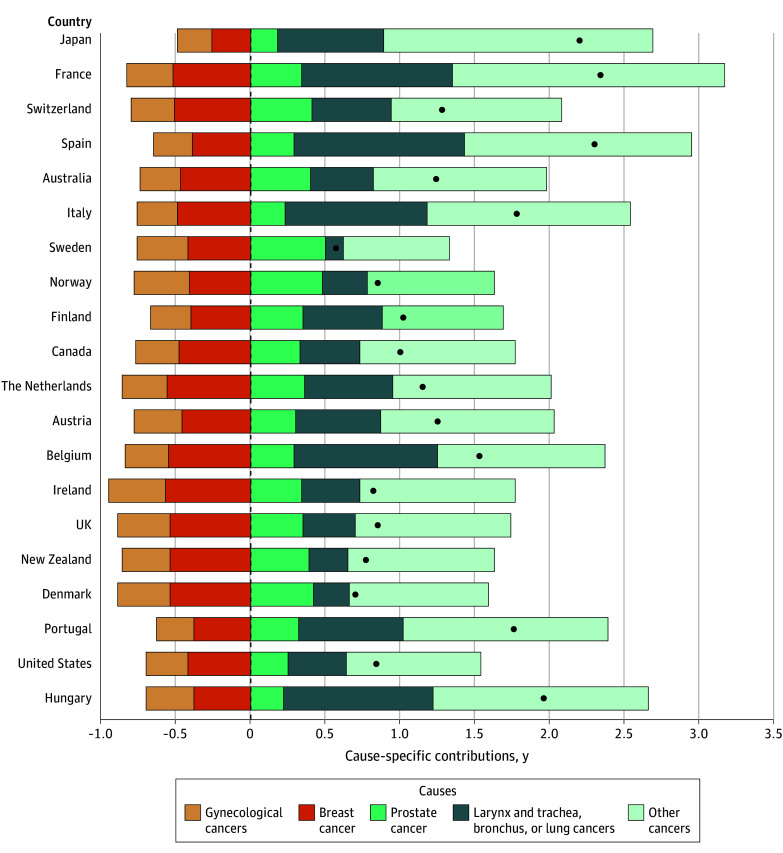
Bar Graph of Estimated Contribution of Female-Specific Cancers and Neoplasms to the Total (All-Cause) Sex Gap in Truncated Cross-Sectional Average Length of Life for Selected Low-Mortality Countries, 1955-2020 Countries are ordered by descending female truncated cross-sectional average length of life values, from the highest (Japan) to the lowest (Hungary). Dots correspond to the contribution (addition of positive and negative) of all cancers to the total (all-cause) sex gap in truncated cross-sectional average length of life (TCAL). TCALs for New Zealand and Portugal correspond to the periods 1955 to 2018 and 1955 to 2019, respectively, due to data availability. Data used are described in eTable 1 in Supplement 1.

Figure 4 summarizes the estimated contribution of female reproductive cancers to the observed sex difference in TCAL across the 20 countries. The contribution ranged from −0.58 (95% CI, −0.61 to −0.55) years in Ireland to −0.27 (95% CI, −0.28 to −0.26) years in Japan for breast cancer, and from −0.38 (95% CI, −0.41 to −0.34) years in Ireland to −0.24 (95% CI, −0.25 to −0.23) years in Japan for gynecological cancers.

In a hypothetical scenario in which all female reproductive cancers were eliminated, the sex gap in TCAL would increase by an estimated mean of 0.77 (95% CI, 0.75-0.78) years, with country-specific estimates ranging from 0.96 (95% CI, 0.92-1.00) years in Ireland to 0.51 (95% CI, 0.50-0.52) years in Japan. All other cancers, including prostate cancer and a combination of lung, larynx, trachea, and bronchus cancers, contributed to a wider survival gap.

The estimated contribution of each of the major causes of death to the TCAL survival gap for each country is shown in eFigure 2 and eTable 5 in Supplement 1. These findings confirm that most of the causes of death contributed positively to the gap, except the female reproductive cancers.

## Discussion

Traditional narratives regarding male disadvantage in longevity often overlook female-specific risks. This cohort study provides new insights into the survival gap for 20 low-mortality countries, using an approach that incorporates historical mortality data. While females maintained a consistent survival advantage over males for all-cause mortality, this advantage was attenuated by an excess in female cancer mortality, which was largely due to female reproductive cancers in midlife. This relative disadvantage was consistently observed between 35 and 60 years of age and remained stable across all birth cohorts and examined countries.

Consistent with previous research, our findings confirmed that major causes of death, such as cardiovascular disease, lung cancer, and external causes of death, are the main contributors to males’ longevity disadvantage relative to females’. This pattern reflects well-documented sex differences in behavioral risk factors, including higher prevalence of smoking, alcohol use, occupational hazards, and violence among males.^14,15,16,17^ However, our results revealed an important countervailing trend: in all countries studied, females born since at least the 1930s have consistently experienced higher mortality from neoplasms than males between the ages of 35 and 60 years. This cohort- and age-specific disadvantage has remained stable over time and is due to the impact of female reproductive cancers. An additional excess in female mortality when compared with their male counterparts is in the respiratory tract or smoking-related cancers (lung, larynx, trachea, and bronchus cancers),^17^ observed in recent years for young female cohorts in Canada, Denmark, the Netherlands, New Zealand, and Sweden (see the eResults in Supplement 1).

Globally, cancer survival has improved significantly in recent decades, largely due to advances in early detection and treatment.^32^ Mortality from female reproductive cancers has declined in many high- and middle-income countries—a trend likely due to multiple factors, including the introduction of population-wide screening programs, improvements in treatment,^33^ and more judicious use of menopause hormonal therapy.^34,35^ Despite substantial progress in cancer control, our findings underscore the persistent burden of breast and gynecological cancers and the continued need for comprehensive efforts in prevention, early detection, and treatment.

The incidence of cancers of the breast, ovary, and uterus rose more sharply during the reproductive years than before puberty and after menopause, highlighting the central role of sex hormones in their cause.^22,23,24,25,26,32,33,34,35,36^ Menopause is considered to confer survival advantages for individuals and the species. Without the onset of menopause, the incidence of female reproductive cancers at older ages would likely be substantially higher. Thus, the elevated risk of breast and gynecological cancers during the reproductive years may be interpreted as a biological cost—or the price of reproduction.

The adjusted median age at diagnosis of breast cancer ranges from 49 years in countries such as the Republic of Korea and Iran to 59 years in the US, Canada, and several European nations.^37^ In most countries, however, the median age at diagnosis of breast cancer is substantially lower than that of other common cancers; this is primarily due to variation in the diagnosis of postmenopausal breast cancer, whereas the diagnosis of premenopausal (early-onset) breast cancer remains relatively consistent across countries—underscoring the influence of hormonal exposures in breast cancer cause at younger ages.^37^

The consistent burden of female reproductive cancers in midlife points to an opportunity for earlier detection and better management strategies targeting reproductive ages. Country-specific differences may be explained through differences in health care access, cancer prevention programs, or reproductive health policies. For example, in countries where early human papillomavirus vaccination programs were implemented, the substantial reduction in cervical cancer incidence is increasingly evident.^38,39^ Countries differ substantially in disease epidemiology, exposure to risk factors, screening practices, treatment availability, and cause-of-death coding. While sex differences in mortality in midlife vary between countries, and the optimal magnitude of difference is not known, cross-national comparisons can provide important insights, including for the development of preventive strategies targeting vulnerable groups.

Methodologically, using TCAL was central for the quantification of the survival gap. TCAL incorporates the historical mortality experience of all birth cohorts alive at a given time and provides a more comprehensive, cohort-sensitive alternative to a period or cohort LE. Sensitivity analysis of TCAL showed minor deviations (<0.23 years) from the results obtained when including data for the period 1955 to 2019 compared with 1955 to 2020, as opposed to the sharp declines reported for life expectancies between 2019 and 2020 (eTable 6 in Supplement 1).^40^ The Lexis surfaces with sex decomposition results further enabled us to visually track the stability and timing of age-specific sex differences in cause-specific mortality over the life course.^24^ The advantages of TCAL become obvious when aiming to capture the effects of treatments, early diagnosis, and interventions, which may have a delayed effect in populations. However, disentangling effects of specific interventions, particularly in international comparisons, would require extensive information for country-specific health policies.

### Limitations

This study has limitations that should be acknowledged. Despite the use of high-quality databases (Human Mortality Database and World Health Organization), differences in death certification and diagnostic coding across time and between countries may affect comparability, especially for cancer subtypes. As a sensitivity analysis, we varied the time series included in the TCAL calculations (eg, starting in 1965 or 1975), which supported the robustness of our findings regarding the presence of a female cancer disadvantage (eResults in Supplement 1). Most of the empirical evidence to date has been obtained in high-income settings, and more research is needed on middle- and low-income countries with difference in reproductive patters, and relatively high child, maternal, and HIV-related mortality, which could reduce or reverse the female advantage in longevity. Additionally, patterns of female cancer mortality and access to health care differ substantially in low- and middle-income countries from those in high-income countries.

## Conclusion

In this population-level cohort study of 20 low-mortality countries, we found that, although females consistently outlived males, their survival advantage was hampered by the burden of female reproductive cancers, particularly in midlife. By analyzing the survival gap with respect to age, birth cohort, and calendar year, we revealed a consistent cross-country pattern of higher cancer mortality among females aged 35 to 60 years compared with males. Further disaggregation analysis supported the association of female reproductive cancers—breast and gynecological cancers—with this female disadvantage. These findings shift attention from the well-established male mortality disadvantage to a less recognized, biologically rooted, female vulnerability associated with reproductive function. Framing this excess risk as the price of reproduction provides critical insight into gender differences in longevity and underscores the need for increased efforts in prevention, early detection, and equitable access to treatment of female reproductive cancers across the lifespan.

## Notes

Supplement 1. eTable 1. Study populations and data availability by data source (as of 15 May 2025) eTable 2. Causes of death and the corresponding *International Classification of Diseases* (*ICD*) codes from *ICD 7* to *ICD 10* eAppendix. Brief description of the truncated cross-sectional average length of life (TCAL) and including uncertainty in TCAL eFigure 1. Lexis diagram showing the data required for period and cohort life expectancies, e_0_, and the truncated cross-sectional average length of life, TCAL. eTable 3. Female and male counts of deaths and exposure used in the truncated cross-sectional average length of life, TCAL(1955, 2020), for selected low-mortality countries eTable 4. Female and male truncated cross-sectional average length of life, TCAL(1955, 2020), and life expectancy at birth in 2020, their sex gap and the difference between measures, for selected low-mortality countries eResults. Sex differences in survival (TCAL_F-TCAL_M) and the contributions of causes of death, selected low-mortality countries between 1955 and 2020. List of countries: Australia, Austria, Belgium, Canada, Denmark, Finland, France, Hungary, Ireland, Italy, Japan, the Netherlands, New Zealand*, Norway, Portugal*, Spain, Sweden, Switzerland, the United Kingdom and the United States eFigure 2. Contribution of major causes of death to the sex gap in TCALs, by selected low-mortality countries eTable 5. Contribution of major causes of death to the sex gap in TCALs, by selected low-mortality countries eTable 6. Female and male truncated cross-sectional average length of life, TCAL, with data to the year of 2020, TCAL(1955, 2020), and to the year of 2019, TCAL(1955, 2019), and their difference, for selected low-mortality countries eReferences

Supplement 2. Data Sharing Statement
