# Supplementary material for: Female Reproductive Cancers and the Sex Gap in Survival
Source: JAMA Netw Open. 2026 Mar 10;9(3):e261256. doi: 10.1001/jamanetworkopen.2026.1256 (PMC12976787; doi:10.1001/jamanetworkopen.2026.1256)
Supplement: Supplement 1. — eTable 1. Study populations and data availability by data source (as of 15 May 2025) eTable 2. Causes of death and the corresponding International Classification of Diseases (ICD) codes from ICD 7 to ICD 10 eAppendix. Brief description of the truncated cross-sectional average length of life (TCAL) and including uncertainty in TCAL eFigure 1. Lexis diagram showing the data required for period and cohort life expectancies, e0, and the truncated cross-sectional average length of life, TCAL. eTable 3. Female and male counts of deaths and exposure used in the truncated cross-sectional average length of life, TCAL(1955, 2020), for selected low-mortality countries eTable 4. Female and male truncated cross-sectional average length of life, TCAL(1955, 2020), and life expectancy at birth in 2020, their sex gap and the difference between measures, for selected low-mortality countries eResults. Sex differences in survival (TCAL_F-TCAL_M) and the contributions of causes of death, selected low-mortality countries between 1955 and 2020. List of countries: Australia, Austria, Belgium, Canada, Denmark, Finland, France, Hungary, Ireland, Italy, Japan, the Netherlands, New Zealand*, Norway, Portugal*, Spain, Sweden, Switzerland, the United Kingdom and the United States eFigure 2. Contribution of major causes of death to the sex gap in TCALs, by selected low-mortality countries eTable 5. Contribution of major causes of death to the sex gap in TCALs, by selected low-mortality countries eTable 6. Female and male truncated cross-sectional average length of life, TCAL, with data to the year of 2020, TCAL(1955, 2020), and to the year of 2019, TCAL(1955, 2019), and their difference, for selected low-mortality countries eReferences [file jamanetwopen-e261256-s001.pdf]

Supplemental Online Content

Canudas-Romo V, Su W, Banks E, Timonin S. The price of reproduction and contribution of female reproductive cancers to the sex gap in survival. *JAMA Netw. Open*. 2026;9(3):e261256. doi:10.1001/jamanetworkopen.2026.1256

eTable 1. Study populations and data availability by data source (as of 15 May 2025)

eTable 2. Causes of death and the corresponding *International Classification of Diseases (ICD)* codes from *ICD 7* to *ICD 10*

eAppendix. Brief description of the truncated cross-sectional average length of life (TCAL) and including uncertainty in TCAL and including uncertainty in TCAL

eFigure 1. Lexis diagram showing the data required for period and cohort life expectancies, e_0, and the truncated cross-sectional average length of life, $TCAL$.

eTable 3. Female and male counts of deaths and exposure used in the truncated cross-sectional average length of life, TCAL(1955, 2020), for selected low-mortality countries

eTable 4. Female and male truncated cross-sectional average length of life, TCAL(1955, 2020), and life expectancy at birth in 2020, their sex gap and the difference between measures, for selected low-mortality countries

eResults. Sex differences in survival ($TCAL_F - TCAL_M$) and the contributions of causes of death, selected low-mortality countries between 1955 and 2020. List of countries: Australia, Austria, Belgium, Canada, Denmark, Finland, France, Hungary, Ireland, Italy, Japan, the Netherlands, New Zealand*, Norway, Portugal*, Spain, Sweden, Switzerland, the United Kingdom and the United States

eFigure 2. Contribution of major causes of death to the sex gap in TCALs, by selected low-mortality countries

eTable 5. Contribution of major causes of death to the sex gap in TCALs, by selected low-mortality countries

eTable 6. Female and male truncated cross-sectional average length of life, TCAL, with data to the year of 2020, TCAL(1955, 2020), and to the year of 2019, TCAL(1955, 2019), and their difference, for selected low-mortality countries

eReferences

This supplemental material has been provided by the authors to give readers additional information about their work.

© 2026 Canudas-Romo V et al. *JAMA Network Open*.

eTable 1. Study populations and data availability by data source (as of 15 May 2025)

	Country name	*HMD data availability*	*WHO data availability*	*Years used for TCAL calculations*
1	Australia	1921 - 2021	1950 - 2023 (excluding 2005)	1955 - 2020
2	Austria	1947 - 2023	1955 - 2023	1955 - 2020
3	Belgium	1841 - 2023	1954 - 2021	1955 - 2020
4	Canada	1921 - 2022	1950 - 2022	1955 - 2020
5	Denmark	1835 - 2024	1951 - 2022	1955 - 2020
6	Finland	1878 - 2023	1952 - 2022	1955 - 2020
7	France	1816 - 2022	1950 - 2022	1955 - 2020
8	Hungary	1950 - 2020	1955 - 2023	1955 - 2020
9	Ireland	1950 - 2022	1950 - 2022	1955 - 2020
10	Italy	1872 - 2021	1951 - 2021	1955 - 2020
11	Japan	1947 - 2023	1950 - 2021	1955 - 2020
12	The Netherlands	1850 - 2022	1950 - 2023	1955 - 2020
13	New Zealand*	1948 - 2021	1950 - 2018[a]	1955 - 2018[a]
14	Norway	1846 - 2023	1951 - 2023	1955 - 2020
15	Portugal*	1940 - 2023	1955 - 2019[b], 2022	1955 - 2019[b]
16	Spain	1908 - 2023	1951 - 2022	1955 - 2020
17	Sweden	1751 - 2023	1951 - 2023	1955 - 2020
18	Switzerland	1876 - 2023	1951 - 2022	1955 - 2020
19	United Kingdom	1922 - 2022	1950 - 2021 (excluding 2000)	1955 - 2020
20	United States	1933 - 2023	1950 - 2022	1955 - 2020

Note: [a] The WHO did not include causes of death counts for New Zealand* for any of the years in the period 2019-2020. [b] For Portugal* the WHO data is not available for 2020. For Australia and UK for the years of 2005 and 2000, respectively, imputation of the causes of death information was based on the average data of the adjacent years.

© 2026 Canudas-Romo V et al. *JAMA Network Open*.

eTable 2. Causes of death and the corresponding International Classification of Diseases (ICD) codes from ICD 7 to ICD 10

Cause of death name	ICD 7	ICD8	ICD 9	ICD 10
Neoplasms	140-239	140-239	140-239	C00-D48
Breast cancer	170	174	174	C50
Gynecologic cancer (cervical, ovarian, uterine, vaginal and vulvar cancers)	171-176	180-184	179-184	C51-C58
Lung/larynx/trachea/bronchus cancer	161-163	161-162	161-162	C32-C34
Prostate cancer	177	185	185	C61
Diseases of the circulatory system	400-468	390-458	390-459	I00–I99
External causes of death	E800-999	E800-999	E800-999	V01–Y89
Other causes (only ICD 10 shown)	Communicable, maternal, perinatal and nutritional conditions (A00-B99, D50-D53, D64.9, E00-E02, E40-E46, E50-E64, G00-G04, G14, H65-H66, J00-J22, N70-N73, O00-O99, P00-P96, U04, U07.1, U07.2, U09.9, U10.9); Diabetes mellitus and endocrine disorders (E10-E14, D55-D64 (minus D64.9), D65-D89, E03-E07, E15-E16, E20-E34, E65-E88); Psychiatric conditions (F01-F99); Neuro conditions (G06-G98 (minus G14), U07.0, X41, X42, X44, X45); Sense organ diseases (H00-H61, H68-H93); Respiratory Diseases (J30-J98); Oral conditions (K00-K14); Digestive diseases (K20-K92); Skin diseases (L00-L98). Musculoskeletal diseases (M00-M99); Genitourinary diseases (N00-N64, N75-N98); Congenital anomalies (Q00-Q99); Sudden infant death syndrome (R95); Ill-defined diseases (R00-R94, R96-R99)			

Source: https://platform.who.int/mortality.

© 2026 Canudas-Romo V et al. *JAMA Network Open*.

eAppendix. Brief description of the truncated cross-sectional average length of life (TCAL) and including uncertainty in TCAL

We employed the truncated cross-sectional average length of life as the primary mortality measure. While conceptually similar to period life expectancy, TCAL differs in that it incorporates historical mortality information for all birth cohorts alive at a given time, rather than being based solely on current-period mortality rates.[3,4]

TCAL(Y_1,t) is defined for a period between two years: *t* is the year for which the measure is estimated, in our illustrations 2020, and Y_1 is the earliest year for the available mortality series, here 1955. *TCAL* is computed as,

$$TCAL(Y_1, t) = \int_0^{\omega} l^c(x, Y_1, t)dx, \qquad \text{(e1)}$$

where $l^c(x, Y_1, t)$ is the life table survival function for cohorts reaching age *x* in year *t*, which were born in year *t-x*, and Y_1 represents the first year with data available for that cohort or its birth year, whichever is more recent. The integral is over all ages, from zero to ω, the oldest cohort present in year *t.* The different data used for life expectancy, cohort $e_{0,c}(1910)$ and period $e_{0,p}(2020)$, as well as for TCAL are illustrated in eFigure 1 with a Lexis surface (a visual representation with ages in the vertical axis, birth cohorts in diagonal lines, and calendar years in horizontal axis).

TCAL summarizes the available cohort mortality history into one measure and its comparison across populations can be disentangled into age- and cohort-contributions, which explain the survival disparity. TCAL remains stable even when data availability for some countries was shorter. For example, in Figure 1, New Zealand (up to 2018) and Portugal (up to 2019) were missing some years of information up to 2020, but as seen in eFigure 1, they represent only one or two years of information for each of the cohorts present at that point. For the rest of the countries included in eTable 1, the first year of excess mortality of COVID-19, namely 2020, was included in the analysis. However, this made little deviations (less than 0.23 year) from the results obtained when including only data for the period 1955-2019, as opposed to the sharp declines reported for life expectancies between 2019 and 2020 (Supplementary eTable 5 includes the TCAL sensitivity analysis). Using mutually exclusive causes of death and associated single decrement techniques it was possible to calculate cause-contribution to TCALs comparisons. From a public health perspective, this approach enables the identification of birth cohorts and age groups, which experienced periods of higher or lower historical mortality, including from specific causes of death.

© 2026 Canudas-Romo V et al. *JAMA Network Open*.

Discrete Approximations

As TCAL condenses the available cohort mortality history into one measure, any difference between TCALs allows for identification of the cohort-specific contributions to the mortality gap. The difference in TCAL*s* between females (F) and males (M) is then

$$TCAL_F(Y_1,t) - TCAL_M(Y_1,t) = \int_0^\omega l_F^c(x,Y_1,t) - l_M^c(x,Y_1,t)dx, \quad \text{(e2)}$$

where $l_i^c(x,Y_1,t)$ is the survival function for the cohort aged *x* at time *t* in population *i,* referring to *i*=female or male. The integral in equation (e2) corresponds to differences between the cohorts from each sex, aged from 0 to ω, and present at time *t*, and where both populations have the same age-specific death rates in years before Y_1. That is, for the years without information, the same imputation is included for both sexes. The latter information before year Y_1, influences the results of TCALs, but the survival gap will be calculated solely based on the available cohort information. Thus, the survival differences on the right side of equation (e2) allows to quantify the mortality contribution of each cohort present in year *t*. The difference between TCAL*s* is comparable to differences in life expectancy in showing the number of years one population is lagging behind another (Supplementary eTable 1). Age- and cohort-contributions, $\Delta(a,t-x)$, to the difference $TCAL_F - TCAL_M$ can be estimated as

$$\Delta(a,t-x) = \left[\frac{l_F^c(x,Y_1,t)+l_M^c(x,Y_1,t)}{2}\right] ln\left[\frac{{}_1p_a(t-x,F)}{{}_1p_a(t-x,M)}\right], \quad \text{(e3)}$$

where ${}_1p_a(t-x,i)$ is the probability of surviving from age *a* to *a+1* for the cohort born in year *t-x* in population *i*. The survival function is the product of all the age-specific probabilities of surviving as, $l_i^c(x,Y_1,t) = \prod_{a=0}^{x-1} {}_1p_a(t-x,i)$. Finally, instead of the integrals in equation (e2), the summation over cohorts and ages of the age-cohort contributions, $\Delta(a,t-x)$, returns the difference in *TCALs*

$$TCAL_F(Y_1,t) - TCAL_M(Y_1,t) \approx \sum_{x=1}^{\omega}\sum_{a=0}^{x-1}\Delta(a,t-x). \quad \text{(e4)}$$

Through decomposition (e4), mortality comparisons between female and male birth cohorts can be obtained. To further include contributions by specific causes of death, the proportion of deaths at age *a* in the cohort *t-x* of cause *j* respect to all deaths, denoted as $r(a,i,j,t-x)$ for population *i,* is used. From these proportions, associated single decrement cause *j*-specific probabilities of surviving from age *a* to *a+1* for the cohort born in year *t-x* in population *i*, or ${}_1p_a(t-x,i,j)$ were calculated as

$${}_1p_a(t-x,i,j) = \left[{}_1p_a(t-x,i)\right]^{r(a,i,j,t-x)}. \quad \text{(e5)}$$

The all-cause probability of surviving from age *a* to *a+1* equals the product of the cause specific probabilities, as ${}_1p_a(t-x,i) = \prod_{j=1}^{n} {}_1p_a(t-x,i,j)$, where there are *n* mutually independent and collectively exhaustive causes of death. Substituting equation (e5) in this product and then in (e3) and rearranging the logarithms converts into the age-, cohort- and cause-specific contribution $\Delta(a,t-x,j)$ to the difference in *TCAL*s,

© 2026 Canudas-Romo V et al. *JAMA Network Open*.

$$\Delta(a, t-x, j) = \left[\frac{l_F^C(x,Y_1,t)+l_M^C(x,Y_1,t)}{2}\right] ln\left[\frac{{}_1p_a(t-x,F,j)}{{}_1p_a(t-x,M,j)}\right]. \quad \text{(e6)}$$

To further calculate cause-eliminated analysis in TCALs, we assign probabilities of surviving of 1, ${}_1p_a(t-x,i,k) = 1$ in equation (e5), to the desire cause eliminated k. The age- cohort- and cause-specific contribution to the difference in *TCAL*s is then obtained for the remaining causes as in equation (e6).

The main limitation of the method is data availability. In principle, we would prefer having as much cohort data as possible. However, for many regions of the world this is not available. Further, in most cases the quality of the data has become better over time, which implies that any measure with a cohort perspective will include some of this quality bias from its older information.

Confidence intervals were calculated through a stratified bootstrap algorithm (1000 iterations): this means that for each iteration, conditional to age and year, death counts by causes of death were resampled using a multinomial distribution and TCAL decomposition for all causes was computed. In this way, we had a complete set of cause-specific decomposition of TCAL differences for each simulation, preserving the constraints of adding to the total difference.

Including Uncertainty in TCAL

The method proposed by Chiang (1984) allows estimating confidence limits for conventional life table quantities and can also be applied to TCAL. The approach is based on generating random age-specific death counts and using them to construct simulated life tables. The number of deaths at each age is assumed to be a random variable following a binomial distribution. As shown by Chiang (1984), the trail size is given by $N_x = D_x/q_x$, where q_x is the life table probability of dying at age *x* and D_x is the observed number of deaths (see also Andreev and Shkolnikov 2010).

Randomly generated age-specific numbers of death Y_x are then used to derive simulated probabilities of dying $Q_x = Y_x/N_x$, which form the basis for constructing simulated life tables. In practice, this approach generates a large number (here, 1000 simulations), each based on a different random realization of death counts. The life table quantities obtained from each simulated life table are sorted and the percentiles of their distribution are used to derive 95% confidence intervals for any life table measure. For each of the cohorts included in TCAL, we simulated 1,000 life tables and derived 95% confidence intervals for this measure.

Confidence intervals for the TCAL age- and cause-decomposition were obtained from 1,000 life table comparisons (females versus males). As described above, randomly generated age-specific numbers of deaths Y_x followed a binomial distribution, while for *n* mutually independent and exhaustive causes of death a multinomial distribution was used to generate age- and cause-specific death counts Y_x^i. This procedure ensures that cause-specific deaths sum to the total number of deaths at each age, such that $Y_x = \sum_i Y_x^i$.

This approach for deriving confidence intervals for TCAL, and its decomposition, was developed by Canudas-Romo et al. (2020) and previously applied by Ni et al. (2021). The small and consistent

© 2026 Canudas-Romo V et al. *JAMA Network Open*.

confidence intervals of our TCAL results, show that small uncertainty is obtained when including all the mortality history of the population present at a given time.

© 2026 Canudas-Romo V et al. *JAMA Network Open*.

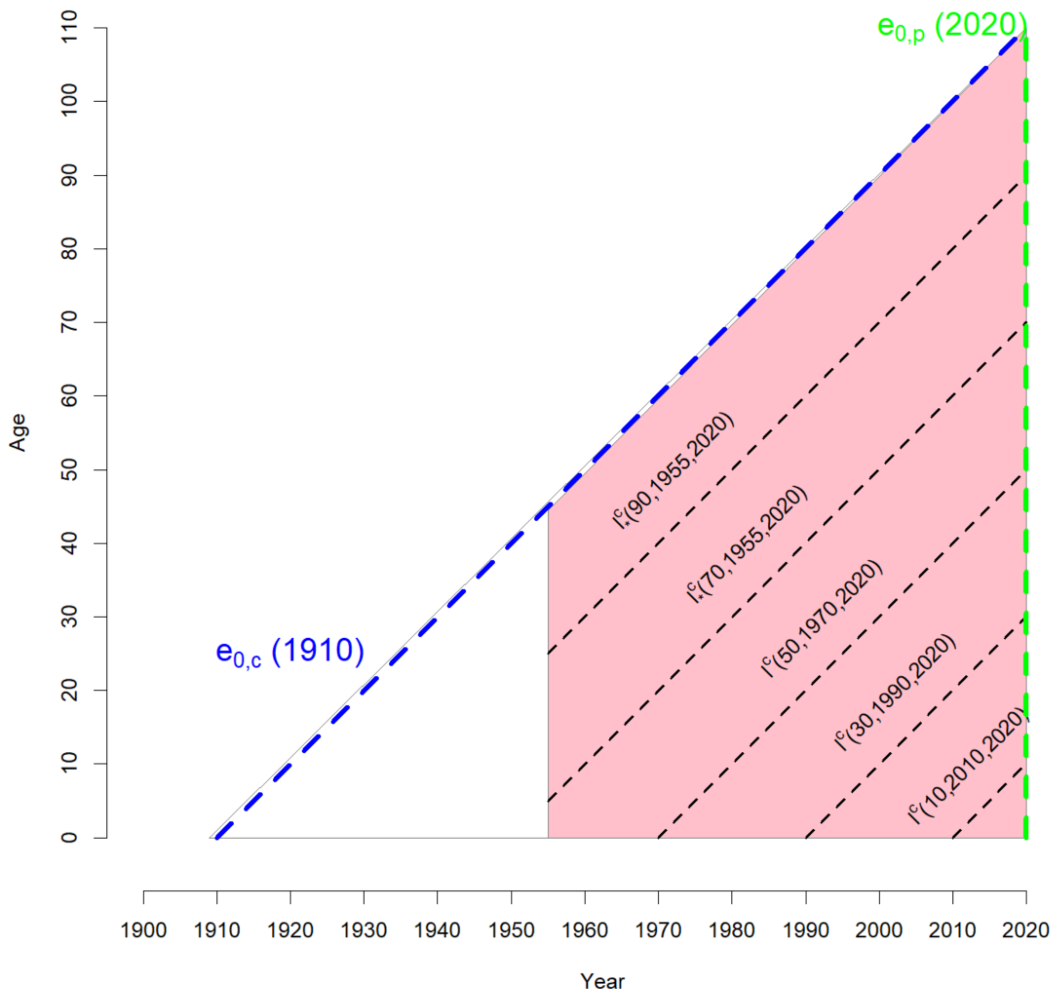

eFigure 1. Lexis diagram showing the data required for period and cohort life expectancies, e_0, and the truncated cross-sectional average length of life, *TCAL*.

Note: $l_*^c(x, Y_1, t)$ are the truncated cohort survival starting their information in the year Y_1 at age $y = Y_1 - (t - x)$ for the cohort born in year $t - x$.

© 2026 Canudas-Romo V et al. *JAMA Network Open*.

eTable 3. Female and male counts of deaths and exposure used in the truncated cross-sectional average length of life, TCAL(1955, 2020), for selected low-mortality countries

Country	Deaths		Exposure		Female Cancer Deaths	
	Females	Males	Females	Males	Count	%
Japan	17,745,510	22,520,173	3,628,265,822	3,515,056,429	1,032,591	5.8%
France	8,765,165	11,568,822	1,688,969,431	1,644,477,696	1,081,035	12.3%
Switzerland	1,052,797	1,246,505	205,823,110	201,405,645	144,115	13.7%
Spain	6,247,578	7,825,405	1,195,556,210	1,168,108,261	503,211	8.1%
Australia	2,316,108	2,975,084	511,603,045	515,534,265	229,925	9.9%
Italy	10,212,857	12,139,542	1,720,444,884	1,650,725,298	1,043,278	10.2%
Sweden	1,521,504	1,790,330	253,966,308	255,479,487	173,744	11.4%
Norway	709,570	853,405	127,821,531	128,906,102	79,575	11.2%
Finland	885,588	1,102,607	153,180,478	148,628,965	81,428	9.2%
Canada	3,903,933	4,870,560	831,617,702	830,080,799	424,031	10.9%
The Netherlands	2,333,196	2,712,999	444,775,844	444,038,609	297,377	12.7%
Austria	1,481,914	1,606,212	237,069,797	224,426,158	179,366	12.1%
Belgium	1,829,179	2,221,879	301,692,788	296,565,057	230,690	12.6%
Ireland	547,149	683,614	110,097,855	110,654,944	61,708	11.3%
UK	10,927,866	12,449,450	1,743,806,394	1,705,588,842	1,350,978	12.4%
New Zealand	505,762	615,811	102,897,679	101,777,616	54,162	10.7%
Denmark	980,763	1,142,606	154,913,861	154,459,037	133,114	13.6%
Portugal	1,972,896	2,444,863	304,010,180	284,931,575	138,079	7.0%
United States	42,675,434	51,435,729	7,703,169,286	7,493,173,664	4,049,533	9.5%
Hungary	2,529,051	3,056,121	316,602,601	297,865,523	233,040	9.2%
Total	119,143,817	145,261,716	21,736,284,808	21,171,883,970	11,520,980	9.7%

Note: countries ordered as in Figure 1. TCALs for New Zealand* and Portugal* were calculated based on the data for 1955-2018 and 1955-2019, respectively, see eTable 1.

Source: data used described in eTable 1.

eTable 4. Female and male Truncated Cross-sectional Average Length of Life, TCAL(1955, 2020), and life expectancy at birth in 2020, their sex gaps, and the difference between measures, for selected low-mortality countries

Country	***TCAL (1955, 2020)***			***Life Expectancy in 2020***			***Difference between LE and TCAL***		
	female	***male***	***sex gap***	***female***	***male***	***sex gap***	***female***	***male***	***sex gap***
Japan	86.21	79.42	6.80	87.75	81.58	6.17	1.54	2.16	-0.63
France	84.77	77.48	7.29	85.15	79.18	5.97	0.38	1.70	-1.32
Switzerland	84.70	79.56	5.14	85.07	81.00	4.07	0.37	1.44	-1.07
Spain	84.61	77.92	6.70	85.01	79.47	5.54	0.40	1.55	-1.16
Australia	84.27	79.42	4.85	85.69	81.63	4.06	1.42	2.21	-0.79
Italy	84.03	78.50	5.53	84.47	79.85	4.62	0.44	1.35	-0.91
Sweden	83.88	79.62	4.26	84.29	80.60	3.69	0.41	0.98	-0.57
Norway	83.75	79.00	4.74	84.90	81.48	3.42	1.15	2.48	-1.32
Finland	83.68	76.67	7.01	84.62	79.03	5.59	0.94	2.36	-1.42
Canada	83.61	78.65	4.96	83.91	79.38	4.53	0.30	0.73	-0.43
The Netherlands	82.99	78.77	4.22	83.08	79.68	3.40	0.09	0.91	-0.82
Austria	82.99	76.90	6.09	83.73	78.93	4.80	0.74	2.03	-1.29
Belgium	82.89	77.19	5.69	83.05	78.53	4.52	0.16	1.34	-1.17
Ireland	82.42	77.76	4.66	83.79	80.00	3.79	1.37	2.24	-0.87
UK	82.41	78.19	4.23	82.37	78.30	4.07	-0.04	0.11	-0.16
New Zealand*	82.41	78.04	4.37	83.62	80.15	3.47	1.21	2.11	-0.90
Denmark	81.66	77.21	4.45	83.51	79.58	3.93	1.85	2.37	-0.52
Portugal*	81.52	74.44	7.08	84.76	78.97	5.79	3.24	4.53	-1.29
United States	81.23	75.84	5.39	79.89	74.34	5.55	-1.34	-1.50	0.16
Hungary	77.85	69.54	8.31	79.02	72.32	6.70	1.17	2.78	-1.61

Note: countries ordered as in Figure 1. TCALs for New Zealand* and Portugal* were calculated based on the data for 1955-2018 and 1955-2019, respectively, see eTable 1.

Source: data used described in eTable 1.

© 2026 Canudas-Romo V et al. *JAMA Network Open*.

eResults. Sex differences in survival ($TCAL^F$-$TCAL^M$) and the contributions of causes of death, selected low-mortality countries between 1955 and 2020

List of countries included in the results-collection: Australia, Austria, Belgium, Canada, Denmark, Finland, France, Hungary, Ireland, Italy, Japan, the Netherlands, New Zealand*, Norway, Portugal*, Spain, Sweden, Switzerland, the United Kingdom and the United States.

Notes: Diagonal dashed lines represent birth cohorts. TCALs for New Zealand* and Portugal* were calculated based on the data for 1955-2018 and 1955-2019, respectively, see eTable 1.

https://vcr1972.github.io/SexGapTCAL

© 2026 Canudas-Romo V et al. *JAMA Network Open*.

eFigure 2. Contribution of major causes of death to the sex gap in Truncated Cross-sectional Average Lengths of Life (TCALs) for selected low-mortality countries

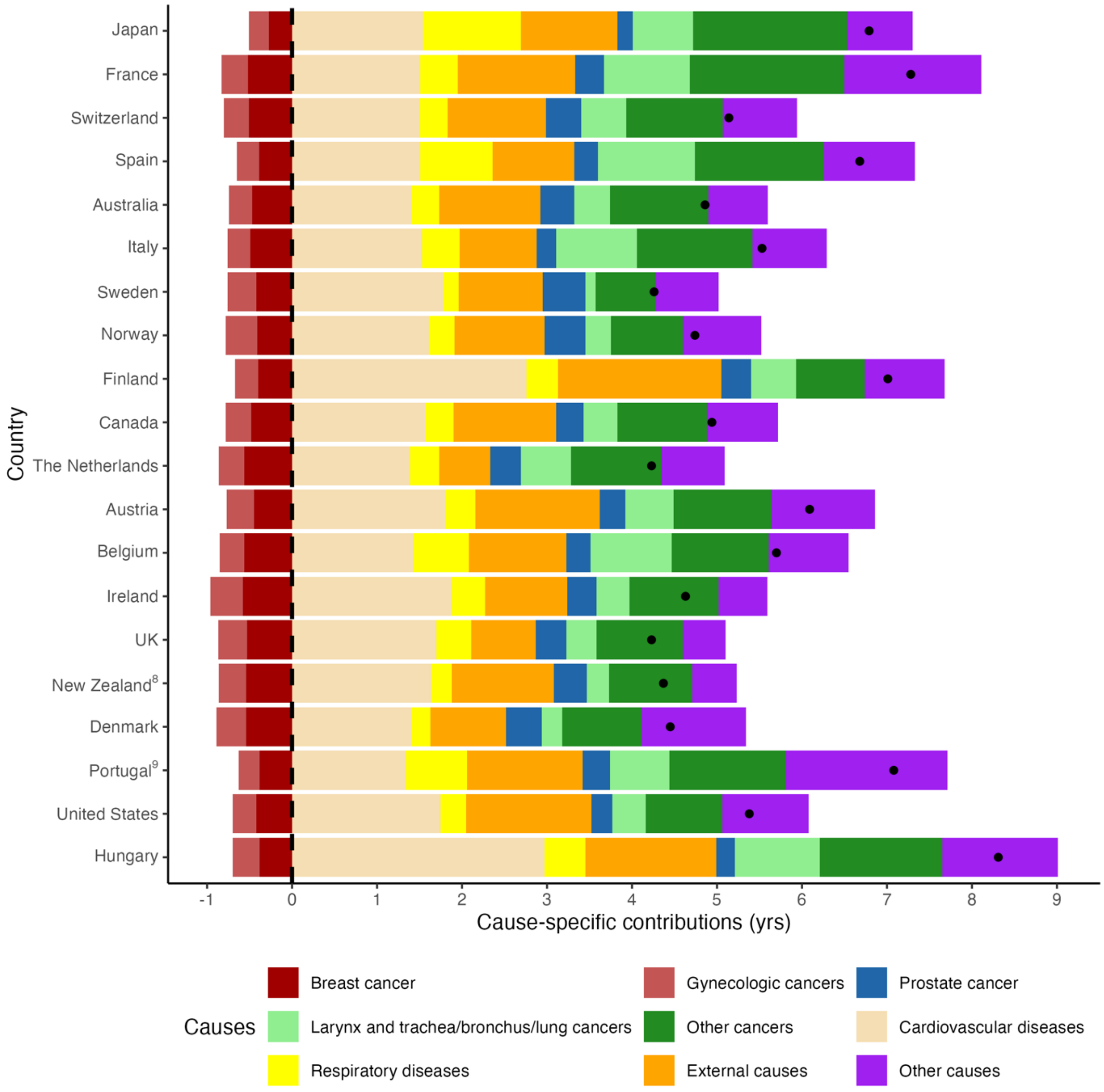

Note: countries ordered as in Figure 1. TCALs for New Zealand[8] and Portugal[9] were calculated based on the data for 1955-2018 and 1955-2019 respectively, see eTable 1.

Source: data used described in eTable 1.

© 2026 Canudas-Romo V et al. *JAMA Network Open*.

eTable 5. Contribution of major causes of death to the sex gap in Truncated Cross-sectional Average Length of Life, TCAL(1955, 2020), for selected low-mortality countries

Countries	*All causes*	*Cardio-vascular diseases*	*Respiratory diseases*	*External causes*	*Neoplasms*	*Breast cancer*	*Gynecologic cancers*	*Female reproductive cancers*	*Prostate cancer*	*Larynx, trachea, bronchus, lung cancers*	*Other cancers*	*Other causes*
Japan	6.79 (6.78,6.8)	1.54 (1.54,1.55)	1.15 (1.15,1.15)	1.14 (1.14,1.15)	2.19 (2.19,2.20)	-0.27 (-0.28,-0.26)	-0.24 (-0.25,-0.23)	-0.51 (-0.52,-0.50)	0.18 (0.17,0.19)	0.71 (0.7,0.71)	1.81 (1.80,1.82)	0.77 (0.76,0.78)
France	7.28 (7.27,7.29)	1.51 (1.50,1.51)	0.44 (0.44,0.44)	1.38 (1.38,1.39)	2.34 (2.34,2.35)	-0.52 (-0.53,-0.51)	-0.31 (-0.32,-0.30)	-0.82 (-0.83,-0.81)	0.34 (0.33,0.35)	1.01 (1.00,1.01)	1.82 (1.80,1.84)	1.61 (1.60,1.62)
Switzerland	5.14 (5.11,5.18)	1.50 (1.49,1.52)	0.33 (0.32,0.34)	1.16 (1.16,1.17)	1.28 (1.27,1.30)	-0.51 (-0.53,-0.49)	-0.28 (-0.30,-0.26)	-0.80 (-0.82,-0.78)	0.41 (0.39,0.43)	0.53 (0.52,0.54)	1.13 (1.09,1.17)	0.87 (0.83,0.91)
Spain	6.68 (6.67,6.70)	1.51 (1.50,1.51)	0.85 (0.85,0.85)	0.96 (0.96,0.96)	2.29 (2.29,2.30)	-0.39 (-0.40,-0.38)	-0.27 (-0.28,-0.26)	-0.66 (-0.67,-0.65)	0.28 (0.27,0.29)	1.14 (1.13,1.14)	1.53 (1.51,1.55)	1.07 (1.05,1.09)
Australia	4.86 (4.83,4.88)	1.41 (1.39,1.41)	0.32 (0.31,0.32)	1.19 (1.18,1.20)	1.24 (1.23,1.25)	-0.47 (-0.48,-0.46)	-0.27 (-0.29,-0.25)	-0.74 (-0.75,-0.73)	0.40 (0.39,0.41)	0.42 (0.42,0.43)	1.16 (1.13,1.19)	0.70 (0.67,0.73)
Italy	5.53 (5.52,5.54)	1.53 (1.52,1.53)	0.44 (0.44,0.44)	0.91 (0.91,0.91)	1.78 (1.77,1.79)	-0.49 (-0.50,-0.48)	-0.27 (-0.28,-0.26)	-0.76 (-0.77,-0.75)	0.23 (0.22,0.24)	0.95 (0.95,0.95)	1.36 (1.34,1.38)	0.87 (0.85,0.88)
Sweden	4.26 (4.23,4.29)	1.78 (1.76,1.79)	0.18 (0.17,0.18)	0.99 (0.99,1.00)	0.57 (0.56,0.59)	-0.42 (-0.44,-0.40)	-0.34 (-0.36,-0.32)	-0.75 (-0.77,-0.73)	0.50 (0.48,0.52)	0.12 (0.12,0.13)	0.71 (0.67,0.75)	0.74 (0.70,0.78)
Norway	4.74 (4.69,4.79)	1.61 (1.59,1.64)	0.30 (0.29,0.32)	1.05 (1.04,1.07)	0.85 (0.84,0.88)	-0.41 (-0.44,-0.38)	-0.37 (-0.4,-0.34)	-0.77 (-0.80,-0.74)	0.48 (0.45,0.51)	0.30 (0.29,0.31)	0.85 (0.80,0.91)	0.93 (0.87,0.99)
Finland	7.01 (6.97,7.05)	2.75 (2.74,2.77)	0.37 (0.37,0.38)	1.92 (1.91,1.93)	1.02 (1.01,1.04)	-0.40 (-0.42,-0.38)	-0.27 (-0.29,-0.25)	-0.67 (-0.69,-0.65)	0.35 (0.33,0.37)	0.53 (0.52,0.54)	0.81 (0.77,0.85)	0.95 (0.90,1.00)
Canada	4.94 (4.92,4.96)	1.57 (1.56,1.58)	0.34 (0.33,0.34)	1.21 (1.21,1.22)	0.99 (0.98,1.00)	-0.48 (-0.49,-0.47)	-0.30 (-0.31,-0.29)	-0.78 (-0.79,-0.77)	0.32 (0.31,0.33)	0.40 (0.40,0.41)	1.05 (1.03,1.07)	0.83 (0.80,0.85)
The Netherlands	4.23 (4.20,4.25)	1.39 (1.38,1.40)	0.34 (0.33,0.35)	0.60 (0.60,0.61)	1.15 (1.14,1.17)	-0.56 (-0.58,-0.54)	-0.30 (-0.32,-0.28)	-0.85 (-0.87,-0.83)	0.36 (0.34,0.38)	0.59 (0.58,0.59)	1.06 (1.02,1.10)	0.75 (0.72,0.78)

© 2026 Canudas-Romo V et al. *JAMA Network Open*.

Austria	6.09 (6.05,6.12)	1.81 (1.79,1.83)	0.35 (0.34,0.36)	1.46 (1.45,1.47)	1.25 (1.24,1.27)	-0.45 (-0.47,-0.43)	-0.32 (-0.34,-0.30)	-0.77 (-0.79,-0.75)	0.30 (0.28,0.32)	0.57 (0.56,0.58)	1.15 (1.11,1.19)	1.22 (1.17,1.27)
Belgium	5.70 (5.67,5.73)	1.43 (1.42,1.44)	0.65 (0.64,0.66)	1.15 (1.14,1.16)	1.53 (1.52,1.54)	-0.56 (-0.58,-0.54)	-0.29 (-0.31,-0.27)	-0.85 (-0.87,-0.83)	0.28 (0.26,0.30)	0.96 (0.95,0.96)	1.14 (1.11,1.17)	0.94 (0.90,0.98)
Ireland	4.66 (4.58,4.69)	1.88 (1.86,1.90)	0.39 (0.37,0.40)	0.97 (0.96,0.99)	0.82 (0.80,0.85)	-0.58 (-0.61,-0.55)	-0.38 (-0.41,-0.34)	-0.96 (-0.99,-0.92)	0.34 (0.31,0.38)	0.39 (0.38,0.4)	1.05 (0.99,1.11)	0.57 (0.50,0.64)
UK	4.23 (4.22,4.24)	1.70 (1.70,1.71)	0.41 (0.40,0.41)	0.76 (0.76,0.76)	0.86 (0.85,0.86)	-0.53 (-0.54,-0.52)	-0.34 (-0.35,-0.33)	-0.87 (-0.88,-0.86)	0.36 (0.35,0.37)	0.35 (0.35,0.35)	1.02 (1.00,1.04)	0.50 (0.49,0.51)
*New Zealand**	4.37 (4.32,4.43)	1.65 (1.63,1.67)	0.24 (0.23,0.25)	1.20 (1.19,1.22)	0.77 (0.74,0.79)	-0.54 (-0.58,-0.50)	-0.32 (-0.35,-0.29)	-0.86 (-0.90,-0.82)	0.39 (0.35,0.43)	0.25 (0.24,0.27)	0.99 (0.92,1.06)	0.51 (0.44,0.58)
Denmark	4.45 (4.41,4.49)	1.41 (1.40,1.42)	0.22 (0.21,0.23)	0.89 (0.88,0.9)	0.70 (0.68,0.73)	-0.54 (-0.57,-0.51)	-0.35 (-0.38,-0.32)	-0.88 (-0.91,-0.85)	0.42 (0.39,0.45)	0.24 (0.23,0.25)	0.93 (0.87,0.99)	1.23 (1.18,1.28)
*Portugal**	7.08 (7.05,7.11)	1.34 (1.32,1.35)	0.72 (0.71,0.72)	1.36 (1.35,1.37)	1.76 (1.74,1.77)	-0.38 (-0.40,-0.36)	-0.25 (-0.27,-0.23)	-0.64 (-0.66,-0.62)	0.32 (0.30,0.34)	0.70 (0.70,0.71)	1.37 (1.33,1.41)	1.90 (1.86,1.94)
United States	5.38 (5.37,5.38)	1.75 (1.75,1.75)	0.30 (0.30,0.30)	1.47 (1.47,1.47)	0.84 (0.84,0.84)	-0.42 (-0.42,-0.42)	-0.28 (-0.29,-0.27)	-0.70 (-0.71,-0.69)	0.25 (0.25,0.25)	0.39 (0.39,0.39)	0.90 (0.89,0.91)	1.02 (1.01,1.03)
Hungary	8.31 (8.28,8.33)	2.97 (2.96,2.99)	0.48 (0.47,0.49)	1.54 (1.53,1.55)	1.96 (1.94,1.97)	-0.38 (-0.40,-0.36)	-0.32 (-0.34,-0.30)	-0.70 (-0.72,-0.68)	0.22 (0.20,0.24)	1.00 (0.99,1.01)	1.44 (1.4,1.48)	1.36 (1.32,1.39)

Note: countries ordered as in Figure 1. TCALs for New Zealand* and Portugal* were calculated based on the data for 1955-2018 and -2019 respectively, see eTable 1. 95% confidence intervals are shown in parenthesis.

Source: data used described in eTable 1.

© 2026 Canudas-Romo V et al. *JAMA Network Open*.

eTable 6. Female and male Truncated Cross-sectional Average Length of Life, TCAL, with data to the year of 2020, TCAL(1955, 2020), and to the year of 2019, TCAL(1955, 2019), and their difference, for selected low-mortality countries

Country	***TCAL (1955, 2020)***		***TCAL (1955, 2019)***		***Difference between TCALs***	
	female	***male***	***female***	***male***	***female***	***male***
Japan	86.21	79.42	86.06	79.23	0.15	0.19
France	84.77	77.48	84.64	77.28	0.13	0.20
Switzerland	84.70	79.56	84.57	79.34	0.13	0.22
Spain	84.61	77.92	84.44	77.71	0.17	0.21
Australia	84.27	79.42	84.11	79.21	0.16	0.21
Italy	84.03	78.50	83.89	78.28	0.14	0.22
Sweden	83.88	79.62	83.73	79.41	0.15	0.21
Norway	83.75	79.00	83.60	78.77	0.15	0.23
Finland	83.68	76.67	83.53	76.44	0.15	0.23
Canada	83.61	78.65	83.48	78.46	0.13	0.19
Austria	82.99	76.90	82.85	76.70	0.14	0.20
The Netherlands	82.99	78.77	82.88	78.56	0.11	0.21
Belgium	82.89	77.19	82.74	76.98	0.15	0.21
New Zealand	82.70	78.42	82.56	78.24	0.14	0.18
Ireland	82.42	77.76	82.21	77.53	0.21	0.23
UK	82.41	78.19	82.26	78.01	0.15	0.18
Portugal	81.72	74.67	81.52	74.44	0.20	0.23
Denmark	81.66	77.21	81.47	76.99	0.19	0.22
United States	81.23	75.84	81.13	75.72	0.10	0.12
Hungary	77.85	69.54	77.71	69.33	0.14	0.21

Note: countries ordered in descending female TCAL values, from the highest in Japan the lowest in Hungary. For this sensitivity analysis using overall mortality in TCALs, data for New Zealand and Portugal were calculated as for the rest of the populations based on the HMD data for 1955-2020 and 1955-2019.

Source: data used described in eTable 1.

© 2026 Canudas-Romo V et al. *JAMA Network Open*.

eReferences

1. Andreev, E. and V. Shkolnikov, Spreadsheet for calculation of confidence limits for any life table or healthy-life table quantity, in MPIDR Technical Report. Max Planck Institute for Demographic Research, (2010).
2. C. L. Chiang. The Life Table and Its Applications. Malabar, FL: Robert E. Krieger Publishing Company, (1984).
3. V. Canudas-Romo, T. Adair, S. Mazzuco, Reflection on modern methods: Cause of death decomposition of cohort survival comparisons. Int. J. Epidemiol. 49, 1712–1718 (2020).
4. V. Canudas-Romo, M. Guillot, Truncated cross-sectional average length of life: A measure for comparing the mortality history of cohorts. Popul. Stud. (Camb.) 69, 147–159 (2015).
5. M. Y. Ni, V. Canudas-Romo, J. Shi, et al., Understanding longevity in Hong Kong: A comparative study with long-living, high-income countries. Lancet Public Health 6, e919–e931 (2021).

© 2026 Canudas-Romo V et al. *JAMA Network Open*.
