# Supplementary material for: Female Reproductive Cancers and the Sex Gap in Survival
Source: JAMA Netw Open. 2026 Mar 10;9(3):e261256. doi: 10.1001/jamanetworkopen.2026.1256 (PMC12976787; doi:10.1001/jamanetworkopen.2026.1256)
Supplement: Supplement 2. — Data Sharing Statement [file jamanetwopen-e261256-s002.pdf]

Data Sharing Statement

Canudas-Romo. Contribution of Female Reproductive Cancers to the Sex Gap in Survival. *JAMA Netw Open*. Published March 10, 2026. doi:10.1001/jamanetworkopen.2026.1256

Data
Data available: Yes
Data types: Other (please specify)
Additional Information: Both databases used in the analysis are free and available online. The Human Mortality Database (HMD) is available online at https://www.mortality.org and the World Health Organization Mortality Database (WHO) at https://www.who.int/data/data-collection-tools/who-mortality-database
How to access data: All codes used in this paper are available in the Github.
When available: With publication

Supporting Documents
Document types: Statistical/analytic code
How to access documents: All analyses were performed in R Studio and R code to replicate our results available at: https://github.com/VCR1972/SexGapTCAL
When available: With publication

Additional Information
Who can access the data: Anyone
Types of analyses: Any
Mechanisms of data availability: R code to replicate our results available at: https://github.com/VCR1972/SexGapTCAL
Any additional restrictions: None
